# The ‘Institutional Lottery’: Institutional variation in the processes involved in accessing late abortion in Victoria, Australia

**DOI:** 10.1016/j.wsif.2023.102822

**Published:** 2023

**Authors:** Casey Michelle Haining, Hilary Bowman-Smart, Anne O'Rourke, Lachlan de Crespigny, Louise Anne Keogh, Julian Savulescu

**Affiliations:** a Centre for Health Equity, Melbourne School of Population and Global Health, University of Melbourne, Victoria, Australia; b Australian Centre for Precision Health, University of South Australia, South Australia, Australia; c Murdoch Children's Research Institute, Victoria, Australia; d Monash Bioethics Centre, Monash University, Victoria, Australia; e Monash Business School, Monash University, Victoria, Australia; f Centre for Biomedical Ethics, Yong Loo Lin School of Medicine, National University of Singapore, Singapore; g Melbourne Law School, University of Melbourne, Victoria, Australia

**Keywords:** Abortion, Local regulation, Access, Lottery, Institutions, Termination Review Committee

## Abstract

Despite abortion being decriminalised in Victoria, Australia, access remains difficult, especially at later gestations. Institutions (i.e. health services) place restrictions on the availability of late abortions and/or require additional requirements to be satisfied (e.g. Hospital Termination Review Committee approval), as a consequence of local regulation (i.e. policies and processes determined at the institutional level). This paper reports on the results of 27 interviews with Victorian health professionals about late abortion processes and the operation of Termination Review Committees in Victorian health services, which were analysed thematically. The results reveal the operation of an ‘institutional lottery’ whereby patients' experiences in seeking late abortion services were variable and largely shaped by the institution(s) they found themselves in.

## Highlights

• Patients seeking late abortions face many access barriers.
• The availability of late abortion in Victoria (Australia) is largely determined at the institutional level.
• There is a lack of transparency around institutional policies and processes with respect to late abortion.

## Introduction

In Australia, abortion is not an uncommon procedure. While abortion data is not routinely and consistently collected, the most recent estimate suggested that the rate of abortion in Australia in 2017–2018 was 17.3 abortions per 1000 patients aged between 15 and 44 years, meaning over 88,000 abortions were performed in 2017–2018 (Keogh et al., 2021). Abortion in Australia is largely regulated at the individual state and territory level (Haining et al., 2022). While abortion is lawful in each jurisdiction, variation exists between states and territories (Haining et al., 2022) and access barriers persist (Millar, 2021a). Barriers are wide-ranging, comprising structural barriers (e.g. affordability, geographic barriers, availability of health information etc.), intrapersonal and interpersonal barriers (e.g. stigma, availability of support networks) and healthcare system specific barriers (e.g. conscientious/institutional objection, availability of willing providers, workforce capacity and workplace capability) (Cleetus et al., 2022; de Moel-Mandel & Shelley, 2017; Sifris & Penovic, 2021).

Patients^2^ may request abortions for a variety of reasons; however, many abortions performed after 20 weeks' gestation will be performed on the grounds of severe fetal abnormalities or maternal illness (Black et al., 2015; Megaw & Dickinson, 2018; Rosser et al., 2022). The provision of safe abortions after 20 weeks is considered crucial in a health system that offers routine screening of the fetus as a core part of obstetric care, especially as most structural abnormalities remain undiagnosed until 19–20 weeks of gestation, unlike aneuploidy which may be diagnosed in the first trimester (Megaw & Dickinson, 2018). Moreover, access to screening may be limited (or delayed) for patients living regionally (Coory et al., 2007) resulting in later diagnoses and patients presenting for abortions at later gestations. Support for late abortions is reflected in community views, with previous research suggesting that most Australians support laws enabling patients to access abortions at later gestations, albeit variation exists depending on circumstances (Cations et al., 2020; de Crespigny et al., 2010).

This article is concerned with the accessibility of late abortion in Victorian health services and aims to describe the variability in institutional policies and processes pertaining to late abortion that exists. Late abortions are medically more complex than abortions performed at earlier gestations (Black et al., 2015; Epner et al., 1998). In Victoria, such abortions will only be accessible at approved health services, which are determined by service capability frameworks set out by the Victorian Government which categorise health services across different levels, defining the scope of health care and the level of complexity of particular health procedures a health service can safely manage based on their level categorisation (DoH, 2019). Such categorisation is based on the health service's workforce skills, infrastructure and equipment, clinical governance, and available support services (DoH, 2019). In addition to this, individual institutions will make policy decisions about the types of services they elect to offer, particularly in relation to ‘contentious’ forms of health care such as abortion. This may include health services claiming an institutional objection on the grounds of their stated institutional values or a policy decision not to offer the service on grounds unrelated to values.

Institutional objection occurs when institutions claim an objector status and compel their employees to refuse to provide lawful health services, such as abortion (Chavkin et al., 2013). Institutional objection in relation to abortion has been identified globally; however, to date, there is limited literature describing the impact of institutional objection on abortion access in Australia (Merner et al., 2023). Despite this, there is evidence that some Australian public maternity hospitals that offer prenatal genetic testing services opt out of providing abortions (Keogh et al., 2019). Despite the paucity of academic literature, institutional objection in the context of abortion has recently been the subject of media and political debate. Indeed, there has been recent media coverage of the impact of institutional objection in the Australian context (Black, 2022; Blau, 2022) and a legislative attempt to restrict the ability of health services to claim an institutional objection within the public health sector in Victoria (SBS, 2022), albeit it ultimately failed (Ward, 2022).

### Regulation of abortion and the late abortion dichotomy

Australian abortion laws, except for the Australian Capital Territory,^3^ have prescribed gestational limits, ranging between 16- and 24-weeks' gestation (Haining et al., 2022). Requests for abortion post such gestational limits require additional legislative requirements to be satisfied for the abortion to be lawfully performed and are subjected to “increased legal and medical oversight and surveillance” (Millar, 2021b, p. 440). The nature of these additional requirements varies across jurisdictions, ranging from requiring women to undergo mandatory counselling to requiring the consensus of two or more medical practitioners^4^ who agree the abortion should be performed in the circumstances (Haining et al., 2022).

Australia's gradualist approach to their abortion laws, emulates the approach of many international jurisdictions (Romanis, 2020). Such a bifurcation has been linked to viability (i.e. the point when the fetus can survive outside the womb) (Romanis, 2020). However, in addition to the concept of viability itself being contested (Halliday et al., 2023), the entrenchment of such a dichotomy, for some, risks perpetuating the stigma typically associated with abortion, including the perception that abortion is equivalent to murder or is a practice that is outside the remit of mainstream health care (Millar, 2021b).

Imposing an arbitrary gestational limit, beyond which abortion becomes inaccessible, has also been scrutinised by professional bodies such as the Royal Australian and New Zealand College of Obstretrics and Gynaecologists (RANZCOG), who perceive using such a “cut-off” as problematic. In their statement on late abortion, the College emphasises that serious fetal abnormalities may not be “identifiable, diagnosed or fully evaluated by the time of an arbitrary gestational age ‘cut-off’” and that “some women have greater difficultly gaining timely access to the necessary specialist services and are particularly vulnerable to missing a gestational age ‘cut-off’” (RANZCOG, p. 2).

#### Regulation of late abortion in Victoria

In the Australian state of Victoria, where this research was conducted, abortion was decriminalised in 2008 via the *Abortion Law Reform Act 2008* (Vic) ‘*ALRA*’. The *ALRA* permits women to access an abortion performed by a medical practitioner^5^ up to 24 weeks' gestation upon request (s 4, *ALRA*). Post 24 weeks, women can access a lawful abortion provided the medical practitioner performing the abortion “reasonably believes that the abortion is appropriate in all the circumstances” and “[consults] at least one other registered medical practitioner who reasonably believes that the abortion is appropriate in all the circumstances” (s 5, *ALRA*). In ascertaining the “appropriateness” of the abortion, the medical practitioners must consider the “relevant medical circumstances” and the woman's “current and future physical, psychological and social circumstances” (s 5, *ALRA*).

While the law sets out the minimum level of requirements that need to be satisfied for the late abortion to be lawfully performed, in practice additional forms of regulation exist. Significantly, for the purposes of this article, is the influence of local regulation that occurs at the institutional level. Indeed, many Victorian health services have included an additional decision-making body known as a ‘Termination Review Committee’ (TRC)^6^ which comprises a multi-disciplinary panel charged with the responsibility of deciding whether the late abortion will be performed.

TRCs have existed in public hospitals in Victoria since 2000 (Woodrow, 2003). The formation of TRCs has been posited to have spurred from a controversial late abortion case^7^ (hereafter ‘the Case’) (Woodrow, 2003). The Case involved a woman (31 weeks' pregnant) who presented at a major Victorian public hospital after her fetus had been diagnosed with skeletal dysplasia (rare genetic disorder that causes abnormal development of bones, joints and cartilage), the woman rejected all other management options and was acutely suicidal (de Crespigny & Savulescu, 2004; Gerber, 2007). The abortion was performed at 32 weeks (de Crespigny & Savulescu, 2004; Gerber, 2007). Several of the medical practitioners plus a genetic counsellor were fired or suspended from their positions. The Case captured the attention of Senator Julian McGauran, who opposed abortion. He reported the practitioners involved to the Medical Practitioners Board. The Case dragged on for years in three courts and was subject to investigations by the hospital, coroner, the police and the Medical Practitioners Board (Nader, 2007). The complaint of professional misconduct was dismissed by the Board as being frivolous and vexatious, and the coronial inquiry deemed that the Case fell outside its jurisdiction (de Crespigny & Savulescu, 2004; Gerber, 2007).

In 2008, the Victorian Law Reform Committee (VLRC) was tasked to consider the decriminalisation of abortion. In doing so, it also considered late abortion and the role of TRCs (VLRC, 2008). Some submissions to the VLRC emphasised that patient autonomy and informed consent should be the only relevant considerations in determining whether an abortion should be performed, and hence the opinions of health practitioners (and indeed TRCs) were perceived as unnecessary (VLRC, 2008). TRCs were also criticised due to their inconsistent membership (and hence inconsistent decision-making), and the lack of transparency around their decisions (VLRC, 2008). Conversely, other submissions were more supportive of TRCs, emphasising that many health practitioners found that late abortions involved “controversial and difficult decisions” and having a consultative decision-making process (such as a TRC) was useful (VLRC, 2008). Ultimately, the VLRC did not recommend that late abortions be considered by a TRC prior to occurring (VLRC, 2008). The VLRC offered three potential models for regulating late abortion (VLRC, 2008). The model ultimately adopted by the Victorian Government which requires two medical practitioners to approve the late abortion prior to occurring (VLRC, 2008). Despite this, many health services continue to rely on TRCs to perform clinical decision-making.

TRCs are a creature of local regulation, and there is currently limited insight into their processes and how they function in practice. In the existing literature, it has been highlighted that TRCs can serve a “protective function” for institutions and clinicians, with some benefits described for patients (Bowman-Smart et al., 2023; Woodrow, 2003). However, the extent to which TRCs do indeed protect patients is a point of contention, with some commentators suggesting that they have instead a gate-keeping effect, causing delays and erecting unnecessary barriers to access (Black et al., 2015; Bowman-Smart et al., 2023).

This paper reports on a subset of findings from a research project which examined Victorian TRCs. It builds on findings previously published which detailed the perceived purposes and functions of TRCs in Victoria (Bowman-Smart et al., 2023), by specifically focusing on late term abortion processes that exist across institutions, the variation that exists and how this may impact on the quality of care delivered.

## Methods

Given the paucity of literature exploring TRCs, a qualitative research design featuring semi-structured interviews was used. We adopted a constructivist paradigm and used a phenomenological methodological approach, permitting participants the opportunity to reflect on their lived experiences with TRCs. Our study design did incorporate the involvement of ‘insiders’ (Kanuha, 2000) given one of the investigators ([LdC]) was involved in the Case. Although, [LdC]'s professional networks (in addition to the networks of other researchers) were relied on, [LdC] was not directly involved in data collection (nor was he one of the participants) or involved in the coding of the data. However, he was involved in the conceptualisation of the project, research design and assisted with the interpretation of the results and drafting the manuscript.

To capture a diversity of experiences, we sought to interview health professionals (including genetic counsellors, midwives and medical practitioners) with direct experience with at least one TRC in Victoria. Purposive sampling was used. Participants were initially identified through researchers' professional networks and contacted by email or phone, and invited to participate in the interview. Due to the reasonably small network of health professionals working in a shared discipline, snowball sampling was used to expand the sample.

### Data collection

Eligible participants took part in a semi-structured interview conducted by one or two interviewers ([HBS], [LAK], [AoR]) between September 2019 and July 2020. Interviews followed an interview guide which explored: participants' experiences of a TRC, perceived role of the TRC, decision-making principles, doctors who object, outcomes of the TRC's decision, and overall impression of the TRC. Interviews were either conducted face-to-face in hospital offices, the homes of the participants or via video conference (Zoom) and ranged between 46 and 107 min. Where possible, the TRC terms of reference were requested from participants and used to triangulate findings.

### Data analysis

Interview transcripts were analysed using thematic analysis as described by Braun and Clarke (2006). Transcripts were uploaded in qualitative data analysis software *N Vivo 12* (QSR International Pty Ltd, 2020), which was used to store and manage the data. A selection of researchers ([LAK], [HBS], [CMH]) first read through the transcripts to familiarise themselves with the data and developed an initial coding framework based on themes emerging from the data. [HBS] and [CMH] then double- coded a selection of transcripts to test the coding framework and test for inter-coder reliability. Following the test coding, the coding framework was then reviewed and refined following discussions between [LAK], [HBS] and [CMH]. [HBS] and [CMH] then divided the remaining transcripts and coded the remaining interview data according to the revised framework. Ongoing analysis and discussion between the researchers during the coding process refined the sub-analyses for each theme. The data relevant to procedural aspects of TRCs and institutional differences have been extracted, analysed and reported here. Data related to the purpose and function of TRCs has been reported elsewhere (Bowman-Smart et al., 2023).

### Ethical considerations

This research was approved by Monash University Human Research Ethics Committee [MUHREC Project 13334]. To protect the confidentiality of participants, quotes have been de-identified and each participant has been assigned a number (e.g. #1). To further protect the confidentiality of participants (and the institutions they work for) when discussing institutions we have assigned generic labels (i.e. Hospital A, B, C etc.). Individual health services have not been assigned a specific code. Consequently, reference being made to Hospital A across the data set is not necessarily referring to the same institution.

## Results

Our results are based on the data obtained from our sample comprising 27 health professionals who had direct or indirect experience (e.g. by referring patients to institutions with a TRC) with TRCs (see Table 1 for demographics). Some participants were able to comment on the operation of TRCs in multiple institutions. The authors consulted at least one participant from each of the six Victorian public hospitals offering late abortions with a TRC (five were based in Metropolitan Melbourne, one regional) and one public hospital that had a TRC used for earlier gestations. Other regional public hospitals were contacted by the researchers to confirm they did not have a TRC in operation. One participant was able to speak from the perspective of a private hospital that has a TRC, and some participants were able to provide insight about institutions that did not offer late abortions and their associated referral processes (which included referring to health services with a TRC).

**Table 1:** Sample demographics.

Demographic		Number
Gender	Female	18
	Male	9
Health professional role	Obstetrician/gynaecologist/maternal-fetal medicine specialist	15
	Psychiatrist	1
	Neonatologist	1
	Clinical geneticist	2
	Genetic counsellor	6
	Midwife	2

We present results in this section relating to institutional variation in the provision of late abortions, focusing on the variation that exists, and how this impacts care delivery from the perspective of our participants, all of whom are health professionals. Firstly, we consider whether late abortions were available at different institutions and, if so, for which indications. We then consider the management of patients seeking late abortion in cases where these services are not provided within the health service. Finally, we consider how patients are managed and the differential operation of TRCs in institutions offering late abortions.

### Is the service available, and if so, for which indications?

**Fig. 1:**
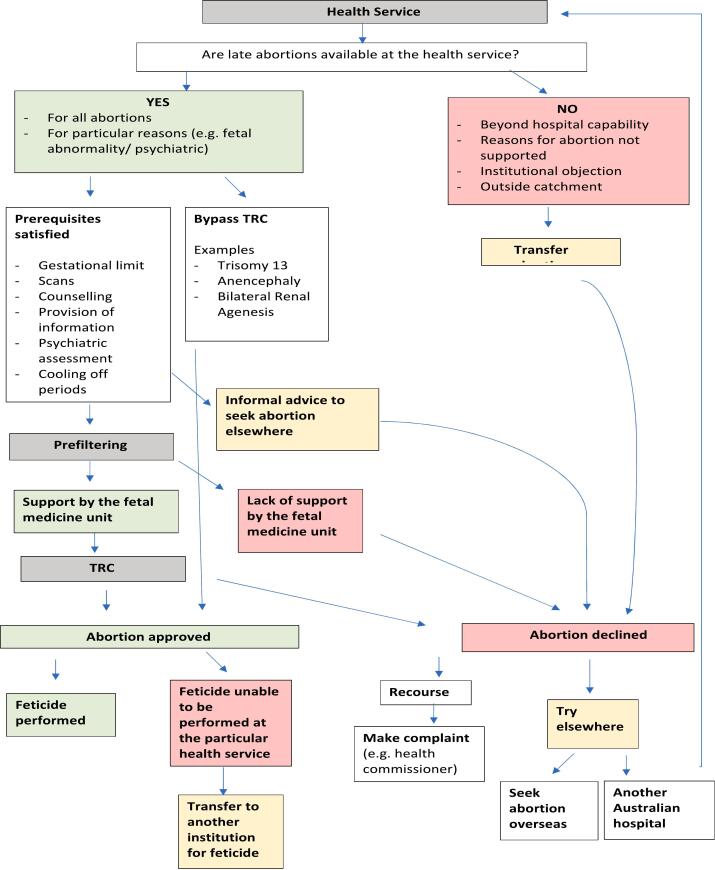
Pathways for obtaining an abortion on fetal abnormality grounds.

Participants revealed that there was variation in the availability of late abortion services across different health services, with late abortions not always routinely available, even in public maternity hospitals offering screening for fetal abnormalities. In this section, we report on the types of restrictions reported, including the influence of the institution's capability, grounds on which the abortion is being sought, and institutional objection to abortion. Fig. 1 provides an overview of the possible pathways for seeking a late abortion on the grounds of fetal abnormality (which, as discussed below, is the grounds that most Victorian health services would be willing to perform a late abortion).

#### Restricting abortion access based on grounds

As the authors have described previously, the availability of a late abortion in Victorian health services was contingent upon the reason in which it was being sought, specifically whether the abortion was being sought on grounds of fetal abnormality or on psychosocial grounds (i.e. any other reason relating to a patient's mental or physical health) (Bowman-Smart et al., 2023).

While most health services would not provide abortions on psychosocial grounds, if there was a psychiatric basis behind the abortion, some health services were prepared to provide late abortions on occasions.

*If a psychiatrist made a recommendation, we would accept that. But if somebody said they didn't want to be pregnant and they wanted a termination, we wouldn't do it.*

[#6]

Outside these exceptions, our data suggests that only one health service was prepared to offer late abortion services on the grounds of fetal abnormality and psychosocial grounds, albeit via two distinct pathways. The existence of the two pathways was thought to be ‘clinically relevant’.

*It's similar, obviously, but it's a different pathway, and that has been set up for a number of reasons. I think clinically it's important that [the fetal medicine unit's] expertise is in the diagnosis and management, counselling, and care of fetal anomalies both structural and genetic, and early onset growth restriction. That obviously has an interplay with the maternal psychological and psychiatric and psychosocial situation, but if the psychosocial situation's the primary reason for wanting a termination, then having an alternative pathway where that's the focus is important to ensure [so] that decision is being made with the required information, and again, freely.*

[#10]

#### Restrictions based on capability of the hospital

Participants described that abortions after a particular gestation were not available in some health services because they were not within the ambit of that health service's capability.

*The capability framework for termination of pregnancy allows [this hospital] … to do up to 24 weeks.*

[#6]

#### Restrictions based on institutional objection

The availability of a late abortion was also influenced by institutional objection. In particular, participants described that an abortion was unlikely to be possible in religiously-affiliated institutions.

*If a woman's been looked after at the [Religious Hospital A] … and they found an abnormality they won't even offer the option of a termination.*

[#23]

*[If you are] referred to [Religious Hospital A] with a significant cardiac condition that may well be lethal …. sorry, we are not in a position to help you, go to [Public Hospital A].*

[#18]

### What are the different ways existing patients are managed when the service they require is not provided?

When a patient is being cared for at a health service that is unable (or unwilling) to provide a late abortion, the patient often needs to be referred to another health service. In some cases, processes were in place to enable this to occur. This section will consider the nature of referral arrangements (where they exist) and reflect on patients' experiences of transfers from the perspectives of our health professional participants.

Participants reflected on the fact that when health services were unable to offer late abortions, referral arrangements were typically in place; however, these were often informal in nature.

*I think it's just a ‘we're not the tertiary hospital, you are, so you need to take them’. I don't know whether it's formal or not.*

[#8]

In the cases of religiously-affiliated institutions, such arrangements were perceived to be clandestine due to theological constraints.

*There's no agreement between us and [Religious Hospital A] to do this. They're not actually allowed to even use the word termination or abortion, so it's completely under the radar. It's something that the doctors there have just come to a non-written understanding historically … we were not allowed to have any MOU or anything at all, but the doctors there do refer people.*

[#6]

*[Religious Hospital A], because of certain theological problems, don't do abortions and also rigorously deny that they ever refer for abortions … [they] have well-oiled, back-door pathways to ensure that people can get a termination.*

[#26]

However, as one participant indicated, such informal arrangement relied on conscientious providers within such institutions to facilitate, whereas some other health practitioners (such as those with a conscientious objection) may not be willing to support such arrangements.

*The need to use backdoor mechanisms means that there is no protection against zealots - it generally works because decent conscientious clinicians do whatever they have to do to get the woman what she needs, but if the doctor you get is a zealot it will not happen.*

[#26]

Even with the existence of such referral arrangements, patients could be disadvantaged based upon where they reside, given some health services would only be willing to take referrals within their catchment, and not take on external patients.

*If you live in [Suburb A] you have to come to this hospital, so if you have a baby with a fetal anomaly you're going to be disadvantaged because you're then going to have to be referred to another hospital, which takes a week or so, and then if [Public Hospital A] reject it, then it could take one to two weeks to [get to Public Hospital B]. The other issue we've had is that [Public Hospital C] … only will look after their own patients … so they'll never accept referrals.*

[#4]

It was also described that referrals were unlikely to be available for patients residing regionally, who will often present to their general practitioner, due to the dearth of local health services.

*She would be quickly sent to [Regional Hospital A] or to Melbourne, if she was lucky … But yeah, if she was unlucky, she would be told she couldn't have it.*

[#13]

#### Experiences of transfers

Participants offered some insight into the transfer experiences of patients and how this inevitably caused delays. It was suggested that the delays were exacerbated by the fact that the patient would need to be ‘worked up’ (i.e. undergo scans and be reviewed by relevant health professionals) again when they entered a new health service.

*It's always tricky because they've always had a very thorough review by the time they've been through us – once they get [here] it … starts again. They have another ultrasound, another obstetric review … review by a paediatrician and a psychiatrist before proceeding to the termination review panel and certainly, the feedback from the women has always been that that's almost quite distressing … it's a check-box exercise in many ways.*

[#9]

In some cases, multiple transfers were necessary before the abortion could be performed.

*I had a patient … that was diagnosed late; she was a [Public Hospital A] patient. She came to [Religious Hospital A to have] her specialist opinion and care. That went for two to three weeks … and then she was beyond [Public Hospital A's capability]… so we sent her to [Public Hospital B], and so she spent two weeks at [Public Hospital B] … They approved her termination, then she went back to [Public Hospital A] … which I just felt was unacceptable.*

[#8]

Participants also reflected on the fact that the need for the patient to be transferred multiple times was damaging to the therapeutic relationship and disrupted the continuity of care.

*[The] whole process is to me problematic … in the sense that, if you have a relationship with a patient you've got the obligation to provide every sort of support that she feels that she needs and she shouldn't have to change horses midstream and move to a [group] of total strangers.*

[#26]

### How much variation is there in the operation of TRCs?

In cases where a patient ended up at a health service that was prepared to consider their late abortion request, prior to the abortion being carried out, their request would need to be considered by a TRC. As previously discussed, TRCs are a product of local regulation and governed by institution-level policies which invites variability across health services. This section will explore some of that variability. In particular, the prerequisites for getting to the committee, the degree in which patient input is accounted for by the committee, and the options available to patients following the committee's decision (or lack thereof) will be considered.

#### Getting to the committee

Before a patient's case would be considered by a committee, there were several steps that needed to be undertaken and criteria that needed to be fulfilled. This section will consider some of those criteria including gestational thresholds, prerequisite assessments and processes, and prefiltering that occurs within the fetal medicine unit.^8^

##### Gestational thresholds

As previously discussed, institutions are bound by service capability frameworks which determine what gestations an abortion can be performed at any given health service, based on the health service's level categorisation. However, some institutions would require patients to be reviewed (and approved) by a TRC after a gestational trigger point which the particular institution would set (most typically 23 and 24 weeks). There were exceptions to this however, whereby particular clinical presentations were permitted to bypass the TRC entirely.

*Fetuses with known lethal conditions, so things like trisomy 13, anencephaly, bilateral renal agenesis, those things don't need to come to a termination review panel.*

[#9]

Despite the presence of these gestational trigger points, participants did indicate that there was scope for ‘controversial’ presentations to be considered by the TRC at earlier gestations.

*There is the option of taking terminations at a lower gestation to the panel if it is felt that the clinician would like the, I guess, imprimatur of the hospital to proceed with something that might be seen as a not straightforward indication for a termination. So, you can ask that one be convened, and normally that would be somewhere between 21 and 23 weeks and five days, not normally at 16 weeks, or something. That sort of 20 to 24 week mark, sometimes people may feel they want to get that official recognition that this is happening and that the hospital would support it.*

[#10]

One participant suggested that there was a public hospital that required a TRC to be convened for all abortions performed at the health service. However, the participant noted that this may be the result of the different function the panel was serving.

*For all of our patients which we send to [Public Hospital A], that hospital will always have a panel, almost irrespective of gestation if they're going for a mid-trimester induction of labour for termination of pregnancy …With [Public Hospital A] it's really, because it's reasonably new work for them and they're not a tertiary centre, I think they just like to make sure the head of the birth suite knows about it … it's almost a communication mechanism.*

[#1]

##### Pre-requisite assessments and processes

Some participants reported that prior to a patient's case being considered by a TRC, several pre-requisites would need to be satisfied. These typically included scans, counselling (psychological or genetic), provision of information, psychiatric assessment and ‘cooling off’ periods.

*When a person first comes with a request, the doctor reviewing them and taking their request looks at all the circumstances; makes sure that all the testing that needs to be done has been done; refers to social work, genetic counselling, [provides] emotional [support and obtains] psych input as necessary; and, asks them to come back in a period of time … [and] gives them some information around the termination review process.*

[#19]

*We require that patients are seen by a psychiatrist before we take it to a termination review panel… for a number of reasons: to have a plan in place for their care afterwards because obviously this is a psychologically traumatic event, no matter how clear the indication; to ensure that they are competent to make this request, and that they are making it of their own free will, and there's no evidence of coercion, and to identify patients who have significant pre-existing, perhaps, mental health concerns that may not have been identified earlier … and make sure that appropriate supports are in place for them, both at the time in the hospital setting, but also in the longer term in the community.*

[#10]

##### Pre-filtering

Unlike abortions being carried out on psychosocial grounds, whereby an alternative pathway was engaged, most abortions being carried out on the grounds of fetal abnormality followed a similar process. In simple terms, a patient would need to satisfy prescribed prerequisites (e.g. undergo counselling, diagnostic scans etc.) and then would have their case discussed by a fetal medicine unit. The fetal medicine unit would then undertake some pre-filtering and decide whether the case would be referred to a TRC. The pre-filtering seemed to be informed by whether the fetal medicine unit felt the abortion would be ‘appropriate’ and whether support could be garnered from staff who would be involved in the abortion.

*If the unit was unanimous in feeling that it wasn't going to be appropriate, then it wouldn't go to the termination review panel because there'd be no point, because one of the things is that it has to be discussed at the [fetal medicine unit] meeting, and there doesn't have to be consensus, but there has to be support. It's quite vague, so it's not like you have to have a quorum plus one or something, but there has to be, essentially, in practice, the majority support for the request.*

[#10]

The inevitable tension between respecting patients' autonomy and the role of the fetal medicine unit was identified. It was highlighted that disagreement at the fetal medicine unit was likely to occur in relation to ‘minor’ abnormalities that were likely to result in minimal disability (if any at all).

*There's a tension there between respecting women's autonomy, and their absolute control over their reproductive health and future, versus also the clarity around the role of termination in the context of a fetal medicine unit and having some sort of sense of it being a pathway for conditions where there is going to be significant disability or suffering. Hypothetically, a termination at 32 weeks for a baby with polydactyly*^9^*on one hand and a normal genetic test, most of us would feel very uncomfortable with that being done on fetal grounds, because it's not really likely to be associated with any substantial concerns in the long term.*

[#10]

Participants also noted that some health service staff would encourage patients to seek an abortion elsewhere if they felt the case would be rejected by the TRC, to avoid further delays.

*The truth is we only take things to TRP here where we're pretty confident that it's going to go through. If there is someone who falls outside of … what our organisation, can do … I would say to them, “I don't think we should move forward with this here, you will have a better hearing at other places.”*

[#19]

Pre-filtering processes, however, were not uniformly reported, with some participants suggesting that all late abortions following the set gestational threshold would be taken to the committee as a matter of process.

*If someone requests termination of pregnancy, to my mind, they can have a termination of pregnancy, if that's what they want. Beyond this particular gestation and in these circumstances, in order to procure the termination, it's a hospital requirement that it be heard at the committee … I don't know of a case where someone has said to them, “I'm not going to present your case,” or, “I'm not going to act on your behalf and request from the hospital.”*

[#25]

*The vast majority of the time, the requests are for the severest end of the spectrum of things, and so in practice, most of the time [it is just a] rubber-stamp[]… because it is actually an important, I think, part of the whole process, to ensure that we try to achieve some sort of consistency of approach.*

[#10]

#### Operation of the TRC and the extent of patient engagement

The nature and composition of the TRCs varied across institutions. Typically, there would be representation from obstetrics and the birth suite/delivery service. However, some institutions had additional representation such as genetics, executive, legal, ethics, social work and medical specialists (e.g. psychiatry, fetal cardiology). Sometimes the meeting of the TRC was pre-determined and would be convened on set days, whereas in other institutions it was convened on a more ad hoc basis and the composition was determined on availability. The composition of the committee and the decision-making principles employed (which will be reported elsewhere) varied across health services. Similarly, the extent to which a patient was permitted to engage with the TRC varied.

Only one participant indicated that the patient meets with the committee.

*They see the patients, if it's a [Public Hospital A] patient they ask the patient to come in and they'll have a consultation with them.*

[#5]

The remaining participants indicated that patients were unable to meet the TRC (outside incidental clinical encounters), and instead the case would be presented to the TRC on their behalf. However, there was some suggestion that the patient could still provide some input (e.g. through writing a letter).

*Sometimes the patients will write a letter and I have encouraged them to do that in the past, because I think the patient voice is very important because otherwise they're relying on us to portray what their reasons are or their feelings and emotions and I think that … firstly, it's a lot of pressure on myself … but also I think it's really important that people hear from the patient.*

[#14]

When participants were probed about why patient input into the committee's decision-making was limited, participants offered several potential reasons why this was the case. Some participants suggested that minimising patient input was intended to 'protect' the patient, the committee members themselves and/or the objectivity of the committee's decision-making.

*There's so many things that are wearing about these circumstances for these patients, but I think having to repeat your story over and over again or feel like this is some sort of exam that you've got to say the right thing to, I think it protects them from that.*

[#1]

*I think just to de-identify the members of the committee. There's no restriction, but it just means protecting people that may not want that exposure to the patient… It might be difficult if, let's say the committee declined … that might be a bit [hard].*

[#7]

*I think that they probably want to keep … the committee objective … and I think that's relatively good, because every story [is] really sad … I think they also want to make sure that the rights of the [fetus are] being … adequately considered.*

[#14]

There was also some suggestion that the TRC was simply an operational requirement (rather than a value judgement on the patient's decision) and hence the patient's input was somewhat irrelevant.

*Thinking about the purpose of the TRP … is not so much to approve the patient's request for a termination of pregnancy, as it is to ensure the organisational support and appropriateness of the termination of pregnancy. So, from that perspective, I guess it was about assessing some of the operational capability of the service, to provide the thing, which is not necessarily relevant to the patient.*

[#9]

##### Post-committee outcomes

Following the meeting of the TRC, the decision would then be communicated to the patient, typically verbally (either over the phone or in person). In some cases, the patient would be given reasons, but this was not uniform.

*Interviewer: So, they don't provide reasons?*

*Interviewee: Not really. Yeah, not that I'm aware of … These people, they really do play God with people's lives, don't they? They can sit there and decide what they think is acceptable to them.*

[#4]

*I believe it would be a verbal communication, and, yes, they would be given the reasons.*

[#20]

###### Accepted requests

In cases where the patient's request for a late abortion was approved, the feticide would be arranged and performed. In some cases, patients would need to have the feticide performed at one hospital and would be managed for delivery at another health service.

*The TRP at [Public Hospital A] will approve it, and if the patient needs feticide or something, that will be done at [Public Hospital A], and then they've sort of pushed [for Public Hospital B] to take those patients back and deliver them as a stillborn.*

[#8]

###### Denied requests

If a patient's late abortion request was rejected by the TRC, some participants indicated that referral to another health service for consideration was technically possible, but this was largely contingent on the institution.

*Interviewer: If a woman were refused, would she be given options to obtain an abortion elsewhere?*

*Participant: We would try, absolutely.*

[#10]

However, whilst the referral was technically possible, many participants indicated that patients may find it difficult accessing a termination if it has already been rejected by one hospital's committee.

*I think that it's unrealistic that they'd get it anywhere else … and I think that that's part of going through the TRC, is that they have to realise that if it is rejected that it's unlikely they'd get it through anywhere else.*

[#14]

There was some indication that patients could seek private providers, but many participants suggested that the majority of private providers would not offer late abortions.

*I don't think it goes beyond 24, so it's not much help for some of these families.*

[#1]

In a similar vein, some participants indicated that some private providers may be reluctant to offer a late abortion if it was refused by a hospital's committee.

*The impression is that private obstetricians out in the community, once the TRC has not approved [the abortion they] feel very uncomfortable about their risk of litigation and bad press if they went against the hospital['s] TRC decision.*

[#16]

There was also indication that patients would seek abortions overseas if they were unable to obtain an abortion locally. If possible (and applicable), some patients would travel back to their 'home country' (e.g. India) to access an abortion.

*[Women would] go back to their home country. They wouldn't do the tourism … they would go back to their home country.*

[#17]

While obtaining abortions privately or overseas was possible, participants indicated that for many of the patients this would not be feasible due to the associated financial burden, especially if it meant travelling somewhere other than one's 'home country' such as the United States.

*Participant: We tell them the availability, but our patients don't have that kind of money.*

*Interviewer: You can't fly to Colorado.*

*Participant: No.*

[#6]

Although not universal, many participants indicated that patients would generally be unable to appeal the decision made by the committee. However, most commonly, in cases of rejection, patients would be provided with alternatives to abortion.

*It's not really an appeal. I guess we've got a specific clause … where [if] a TRP has declined the patient's request for [a termination of pregnancy], alternative clinical treatment options and details of counselling and other support services should be offered to the patient, with sufficient information to allow them to seek alternative options to a [termination of pregnancy].*

[#9]

While an appeal might not be possible, some participants indicated that there was nothing to stop patients from filing a complaint.

*Plenty of avenues for complaint, but none set in stone. A woman could go to a consumer advocate, health commissioner, all sorts of people, but there is no instant right of appeal of a decision.*

[#17]

## Discussion

This paper considered the different institutional processes adopted by Victorian health services relating to the provision of late abortions. While the concept of a postcode lottery whereby variations in access to health care exist based on a geographical area (Graley et al., 2011) is well described, this paper identifies another type of lottery in operation which the authors define as an ‘institutional lottery’. In this sense, the experience of patients seeking a late abortion will vary considerably depending on the institution (i.e. health service) they find themselves in. While the impact of the institutional lottery is likely to be more pronounced in regional and rural communities, with some suggestion from our participants that patients living in such communities are only likely to obtain an abortion if they were 'lucky',^10^ the impact of the institutional lottery also extends to patients living in metropolitan regions.

Given abortion law applies uniformly across Victoria, the source of variation resulting in the institutional lottery was largely the product of local regulation, this includes dedicated policies with respect to late abortions as well as formal processes such as the use of TRCs (which often had terms of reference). However, consistent with findings of previous research, participants identified that institutions had informal (and often hidden) processes. These processes included 'under the radar' referral pathways, which typically exist in religious institutions where there are complicity concerns (Hasselbacher et al., 2020; Stulberg et al., 2016), in addition to pre-filtering practices adopted by fetal medicine units.

In addition to institutional restrictions on the provision of later abortions (either at all, or up to particular gestations), institutions appeared to make policy decisions which restricted the accessibility of a late abortion based on the grounds in which it was being sought (i.e. on fetal abnormality or psychosocial grounds)(Bowman-Smart et al., 2023). Participants described that most non-religiously affiliated public health services in Victoria made policy decisions to not offer late abortions for psychosocial reasons, limiting the availability of late abortions to fetal abnormalities (and psychiatric cases in some circumstances). Such a bifurcation reflects a perennial tendency (which is not limited to the Australian context) to preference ‘medical’ or ‘fetal grounds’ over other reasons for seeking abortion, despite such a distinction not being reflected in law (Kimport et al., 2016; Watson, 2018). The differential treatment of abortions based on the grounds the abortion was being sought is hard to justify clinically given the abortion is not necessarily performed differently. Rather such a distinction seems to reflect a moral judgement on a patient's reasoning for undergoing an abortion masquerading as medical discretion. This type of medical practice perpetuates stigma associated with abortions sought on psychosocial grounds and significantly disadvantages patients who are often already facing challenges that contributed to the need for a late abortion (Erdman, 2017; Watson, 2018). Such a dichotomy is also hard to justify from an ethical perspective with some ethicists arguing that there is no ethical basis to this distinction (Savulescu, 2001). While the discussion of decision-making principles will be explored in a subsequent paper, the appeal to 'fetal interests' and the potential 'comfortableness' of members of the committee is problematic in absence of an agreed account of what these are, and how they weigh against patients' interests. The law in Victoria is silent on fetal interests, and in ethics, some commentators posit that patients' interests should trump those of the fetus (e.g. Thomson, 1971; Isaacs, 2003). Moreover, restricting late abortions purely on the grounds of fetal abnormalities can be construed as discriminatory.^11^ Some commentators argue that the availability of late abortions should be available for a broad range of reasons and not be limited to fetal abnormalities to promote reproductive autonomy and avoid undervaluing people living with a disability (Tongue, 2022). Indeed, it has been argued that limiting abortions to those with fetal abnormalities could institutionalise killing fetuses (particularly those with major abnormalities), and is a form of eugenics (or at least has the same effect) (Savulescu, 2001).

Invoking institution-wide policies which restrict the availability of abortion (or restrict availability based on the grounds it is being sought) significantly impacts access and exacerbates the power imbalance weighted against patients. While individual conscientious objection is protected by law, and it is recognised that not all practitioners will be comfortable providing abortion services (either at all or in particular circumstances), institution-wide restrictions are likely to impede access far more than conscientious objection claimed by individual doctors (Fiala & Arthur, 2017). Consistent with previous findings, many participants' moral position did not align with their institution's policies pertaining to late abortion (Hasselbacher et al., 2020). Indeed, some felt unduly restricted and frustrated by such policies as they were prevented from delivering clinically acceptable care to their patients as a result. Some participants described that the need to transfer patients caused delays in provision and imposed many burdens on patients including those related to cost, travel and discontinuity of care, consistent with burdens described previously (Hasselbacher et al., 2020). This is particularly problematic given participants described that, in some cases, multiple transfers were required, and the patient would be required to undergo unnecessary tests and appointments again. In some cases, the abortion procedure itself was conducted over two institutions (one for the feticide and one for the delivery).

There was also evidence of institutional variation in the extent in which TRCs would adhere to basic principles of procedural fairness and exhibit rigour in their processes, which reflect previously described concerns about clinical ethics committees more broadly (Sokol, 2020). Procedural fairness in health care is assessed by the perceived fairness of the process independent of the outcome and is often characterised by processes viewed to emphasise dignity, promote neutrality and enhance a sense of trust between the patient and the decision-makers (Murphy-Berman et al., 1999). The lack of procedural fairness in some health services created a significant power imbalance in decision-making power in favour of the TRC, leaving patients limited scope to influence the outcome, despite ultimately having to wear the long-term effects of any decision made by the TRC. For example, in some institutions, following a degree of pre-filtering at the fetal medicine unit, if it was decided that there was not enough support amongst the unit, the patient's case would not proceed to the TRC. This was not uniform, however, as in other health services the patient's case would proceed regardless of the initial level of support as a matter of process, even with respect to conditions that would ultimately be 'rubber-stamped'.

The power imbalance was further demonstrated by the fact that only one participant suggested that the committee would hear from the patient directly (although sometimes the committee would consider letters). The limited scope for patients to participate in TRC decision-making processes may amount to a form of epistemic injustice which often permeates health care and describes occurrences where patients' views are discredited (Ho & Unger, 2015). Some participants did contend that the TRC's decision-making process was not intended to limit patients' voices or impose moral judgements on their decisions. However, it is difficult to ascertain to what extent patients' viewpoints are accounted for in the TRC's decision-making if they merely presumed on behalf of the patient rather than being directly canvassed (McLean, 2007).

The lack of adherence to basic procedural fairness principles was also displayed by the tendency for health services to not provide reasons for their decision and to offer limited options for appealing a TRC's decision or complain about the process. While there was opportunity for patients to engage in generic health complaints processes, many participants cast doubt on the efficacy of these mechanisms. Significantly, patients also had very limited options to access a late abortion, especially following a denial from a TRC, which was exacerbated by the monopoly these institutions hold over late abortion services. As many participants described, there was a general sense that if a patient was rejected from a TRC they would find it difficult to be accepted by another institution's TRC, and are unlikely to be supported by a private obstetrician. Patients with the financial means to do so have the option of travelling internationally to access abortion services (Reagan, 2019). Although, as our participants described, this would not be financially viable for many patients, especially if they were unable to go to their 'home country' to access an abortion.

Despite the widespread existence of TRCs in Victorian hospitals offering late abortion, there is limited publicly available information about their processes and a lack of transparency around their operation. Previous international studies have found that patients are not always aware of differential service provision across institutions (Guiahi et al., 2014; Stulberg et al., 2019; Wascher et al., 2020), particularly with respect to the extent in which religious doctrines can curtail the breadth of health services on offer (Stulberg et al., 2019). Such knowledge disparity is compounded by the lack of transparency and ambiguity demonstrated by some institutions with respect to the health care they offer, which has been found to exist in both religious and non-religious institutions (Schwandt et al., 2018). Furthermore, while many religious institutions may be restricted by theological constraints, as some participants indicated, such institutions may still be willing to facilitate a patient's abortion request to some extent. Albeit this was often done in a clandestine manner and the efficacy of such processes was ultimately contingent upon the existence of conscientious providers within the institution to facilitate. Accordingly, even institutions which may be perceived as objecting institutions by patients (e.g. due to their religious affiliation) may still facilitate requests to some extent despite not advertising this fact.

Opaqueness exhibited by institutions in relation to their willingness (or not) to provide (or facilitate access to) particular forms of health care means that patients will only learn about a health service's policies and processes upon admission. This makes it harder for patients to navigate the system and make informed decisions about what institutions to attend. Accordingly, greater transparency around the processes involved in accessing abortions, including how TRCs operate, and the restrictions imposed by institutions are required to assist patients to make informed decisions about the institutions they attend and minimise the impact of the institutional lottery.

### Limitations

Despite efforts to ensure rigour in the research design, this research does have some inherent limitations and accordingly the results should be interpreted in the context of such limitations. The purposive sampling approach meant that some of the participants' perspectives may have been skewed given some participants requested to be interviewed and, in some cases, had a personal relationship with one of the researchers. To minimise potential bias relating to personal relationships, the data analysis was conducted by researchers without relationships with the participants. Moreover, the data reported here pertains to the differential experience of patients based on an institutional lottery but does not include perspectives from patients themselves and rather relies on health professionals' accounts of patients' experiences. Further research which invites patients to share their experiences is warranted. Finally, given the research was done in the Victorian context, the results may have limited generalizability to other settings.

## Conclusion

Despite the liberalisation of abortion laws in Australia, a plethora of barriers remain, particularly when abortions are sought at later gestations. This research has uncovered the impact of institutional policies and processes pertaining to late abortion on Victorian patients, an aspect of abortion care that is largely unknown to the public and opaque, even to patients who find themselves needing a late abortion. Patients seeking late abortions are heavily dependent on institutions to help fulfill their requests. However, as this research reveals, institutional policies and processes related to accessing late abortions in hospitals in Victoria vary by institution subjecting patients to an ‘institutional lottery’, with some institutions erecting greater barriers to access than others. While late abortion for fetal abnormality is offered by some of the hospitals represented, only one hospital offered late abortions for psychosocial indications. The results highlight not only the variability patients will face, but also the complexity of the processes (demonstrated in Fig. 1), the lack of input and control patients have over decision-making that profoundly affects them and the limited options (some of which may not be feasible), and avenues for recourse in cases when a patient's late abortion request is denied. These results reflect the significant power imbalance that exists between patients, health practitioners and institutions, and how this impacts patients' access to abortion and quality of care received. While this research has provided some insight into the impact of institutional practices, further research with patients who have experienced this process should be conducted to gain a greater insight into patients' experiences of the TRC process and to develop recommendations to improve current institutional practices.

## Funding

Research conducted at the Murdoch Children's Research Institute was supported by the 10.13039/501100004752Victorian Government's Operational Infrastructure Support Program. This research was funded in whole, or in part, by the 10.13039/100010269Wellcome Trust [Grant number WT203132/Z/16/Z].

## Declaration of competing interest

One author, LDC, was involved in the Case discussed in this paper. He was not involved in the collection or analysis of the data. JS is a Partner Investigator on an Australian Research Council (grant number LP190100841) which involves industry partnership from Illumina. He does not personally receive any funds from Illumina. JS is a Bioethics Committee consultant for Bayer.

## Footnotes

2 This article largely uses the gender-neutral term ‘patient’ acknowledging that abortions are not limited to one gender identity. The exception to this is when reference is being made to other sources (e.g. participant interviews, empirical studies, policy and legislation) whereby the authors adopt the language used in the source.

3 In the Australian Capital Territory there is no gestational period set out in legislation. However, practically patients seeking abortions post 16 weeks' gestation will be required to travel interstate to access a termination due to capabilities of the institutions within the territory. See ACT Government, Abortion in ACT (Web Page, 17 June 2022). https://www.health.act.gov.au/services-and-programs/sexual-health/abortion-access.

4 In Western Australia this is in the form of a panel that is ministerially appointed.

5 Provisions relating to other health practitioners can be found in sections 6 and 7 of the *ALRA.*

6 This article uses the term ‘Termination Review Committee’ (TRC) throughout for consistency. The authors note that such committees may also be described by different terminology e.g. Termination Review Panel.

7 One of the authors of this paper (LdC) was a medical practitioner involved in the Case.

8 The fetal medicine unit delivers multi-disciplinary care for complex pregnancies which may include obstetric management, pediatric advice, imaging and genetics support.

9 Condition in which a baby is born with one or more extra fingers.

10 This would typically only be available in some metropolitan health services willing to take on rural and regional patients.

11 This argument has formed the basis of recent legal challenges to Abortion Law in the United Kingdom by Heidi Crowter and Máire Lea-Wilson (on behalf of her son Aidan), which has been subject to recent attention (see Tongue, 2022).
